# Efficacy and safety of sacubitril/valsartan vs. valsartan in patients with acute myocardial infarction: A meta-analysis

**DOI:** 10.3389/fcvm.2022.988117

**Published:** 2022-08-24

**Authors:** Pei Yang, Yang Han, Cheng Lian, Xinlei Wu

**Affiliations:** ^1^Department of Cardiology, Tangdu Hospital, Air Force Medical University, Xi’an, China; ^2^Department of Cardiology, Xi’an International Medical Center Hospital, Xi’an, China

**Keywords:** acute myocardial infarction, angiotensin-receptor neprilysin inhibitor, sacubitril/valsartan, cardiac reverse remodeling, meta-analysis

## Abstract

**Background:**

The angiotensin-receptor neprilysin inhibitor (ARNI) sacubitril/valsartan was shown to be superior to the angiotensin receptor blocker (ARB) valsartan in terms of reversing heart failure classification (NYHA classification), reducing N-terminal pro-brain natriuretic peptide (NT-proBNP) level and cardiovascular mortality in many studies. Yet, the efficacy of ARNI did not come from patients with acute myocardial infarction (AMI).

**Methods:**

We searched databases for research published from inception to July 29, 2022, that reported cardiac reverse remodeling (CRR) or security indices. Two reviewers independently screened literature, extracted data, and assessed the risk of bias. Nine studies enrolling 1,369 patients were included to perform a meta-analysis. There were 716 patients in the ARNI group and 653 in the ARB group.

**Results:**

ARNI outperformed ARBs in terms of CRR indices, with striking changes in left ventricular ejection fraction (EF) (MD: 4.12%, 95%CI: 2.36, 5.88, *P* < 0.0001), diameter (MD: –3.40 mm, 95%CI: –4.30, –2.94, *P* < 0.00001, *I*^2^ = 0%) and left atrial diameter (MD: –2.41 mm, 95%CI: –3.85, –0.97, *P* = 0.001, *I*^2^ = 0%), other indices there showed no significant improvements. The incidences of major adverse cardiac events (RR: 0.47, 95%CI: 0.34–0.65, *P* < 0.00001, *I*^2^ = 0%), the heart failure (RR: 0.37, 95%CI: 0.23–0.61, *P* < 0.0001, *I*^2^ = 0%), readmission (RR: 0.54, 95%CI: 0.36–0.80, *P* = 0.003, *I*^2^ = 29%) in the sacubitril/valsartan group were lower than the ARB group, while the incidences of cardiac death (RR: 0.56, 95%CI: 0.28, 1.09, *P* = 0.09), the myocardial infarction (RR: 0.83, 95% CI: 0.39, 1.77, *P* = 0.63), adverse side effects (RR: 1.67, 95% CI: 0.89, 3.13, *P* = 0.11) showed no difference.

**Conclusion:**

This research indicated that early initiation of sacubitril/valsartan in patients after AMI was superior to ARBs in reducing the risks of major adverse cardiac events, heart failure, readmission, and enhancing left ventricular EF, decreasing diameter, left atrial diameter. As for the other outcomes (the incidences of cardiac death, myocardial infarction, and adverse side effects), sacubitril/valsartan demonstrated no obvious advantage over ARBs.

**Systematic review registration:**

https://www.crd.york.ac.uk/prospero/, identifier [CRD42022307237].

## Introduction

Acute myocardial infarction (AMI) poses a serious threat to the health of people because of its high incidence and poor prognosis (1). The principle of therapy is to protect and improve the patient’s cardiac function, save the dying myocardium, and reduce the infarct area. At the same time, actively prevent and treat possible complications. Immediate revascularization in patients suffering from AMI and timely drug treatment are necessary measures to reduce mortality. Improving the prognosis of patients with myocardial infarctions, extending life expectancy, and improving quality of life will be the ultimate objectives of treatment. Angiotensin-converting enzyme inhibitors (ACEI) and angiotensin receptor blockers (ARB) have been widely recognized by clinicians in the cure of patients with AMI and heart failure due to their significant improvement of cardiac function and survival rate (2–4).

Current research has shown that, compared with the patients with heart failure receiving ACEI/ARB, sacubitril/valsartan can inhibit natriuretic peptide system degradation and inhibit the RAAS system (5–7), thus reversing heart failure classification (NYHA classification), significantly reducing the NT-proBNP level, improving exercise tolerance, heart function and inhibiting ventricular remodeling (8, 9), also can significantly reduce the risk of cardiovascular mortality and readmission in patients with heart failure (10–12). Currently, it has been listed as a category I recommendation in heart failure guidelines in Europe, America, and China.

However, the significant efficacy of sacubitril/valsartan only came from patients with heart failure, and the indications do not include patients with AMI. Likewise, the clinical data on the postoperative application of sacubitril/valsartan in patients with AMI are comparatively few (13), and the results are still contentious (13–15). Several studies have demonstrated that sacubitril/valsartan is better and safer at reducing mortality in animals (16–19). Consequently, our current meta-analysis intended to clarify the efficacy and safety of sacubitril/valsartan following percutaneous coronary intervention for patients with AMI.

## Methods

The meta-analysis was performed based on the Cochrane handbook for systematic reviews. The results of this study were arranged based on the Preferred Reporting Items for Reporting Systematic Reviews and Meta-analyses (PRISMA) (20). The data, methods, and materials of this study are available to others for purposes of reproducing the results or replicating procedures by contacting the corresponding author.

## Search strategy

Databases including PubMed, Embase, Cochrane Library, Web of Science, Clinicaltrials.gov, CBM, CNKI, WAN FANG, VIP, and others were searched for relevant studies from inception to July 29, 2022. There were no language limitations in the research. The search strategy is presented in Supplementary Material. The search terms were as follows: myocardial infarction, cardiovascular stroke, myocardial infarct, heart attack; sacubitril/valsartan, sacubitril/valsartan sodium hydrate, LCZ696, endopeptidase, neutral endopeptidase, angiotensin-receptor neprilysin inhibitor (ARNI); ARBs, angiotensin receptor antagonists, angiotensin II receptor blockers, ARB. All searches were performed independently by two reviewers (P. Y. and X.-L.W.) to avoid missing relevant studies. Discrepancies between reviewers were resolved by debate or by a third reviewer.

## Selection criteria

**FIGURE 1:**
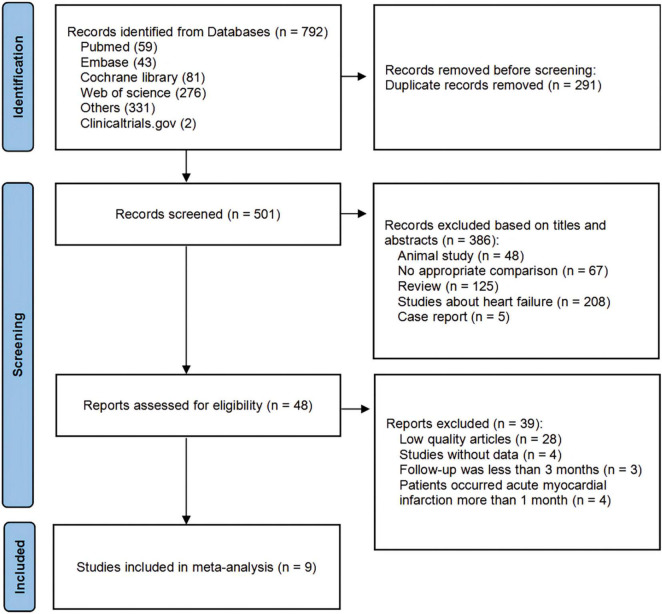
PRISMA (Preferred Reporting Items for Systematic Reviews and Meta-Analyses) flow diagram showing a detailed study selection process.

Studies were screened based on the PICOS criteria (21). We also included the following terms: (1) adult patients (> 18 years) with myocardial infarction; (2) patients assigned to ARNI therapy orally; (3) patients with baseline and follow-up data for at least 1 CRR index, measured by echocardiography; and (4) follow-up for at least 3 months. All publications that met the above criteria were included. The exclusion criteria were as follows: (1) No appropriate comparison; (2) letters, case reports, reviews, and protocols; (3) animal experiments (4) studies about heart failure not after MI; (5) low-quality articles. All titles, abstracts, and full articles were screened by two reviewers (P. Y. and X.-L.W.) to identify the final included studies. In the event of multiple articles reporting the same study, the article with the most complete data was used. Disagreements were resolved by consensus discussion. The search strategy and exclusion criteria are presented in Figure 1.

## Data extraction

Data extraction from included articles was performed by two reviewers (P. Y. and X.-L.W.) respectively. The following data were extracted: first author name, study publication year, study type (RCT, cohort study), patient characteristics (gender, age, medication), sample size, treatment in the control group, and follow-up time. Then dichotomous indices (incidences of MACE, heart failure, readmission, cardiac death, myocardial infarction, adverse side effects, etc.) were extracted. Cardiac reverse remodeling (CRR) indices that directly reflect changes in cardiac structure, including indices of LV volume and dimension [LVEF, end-diastolic diameter (EDD), end-diastolic volume (EDV), end-systolic volume (ESV)], and indices of atrial remodeling [left atrial dimension (Lad)]. Biological indicators (NT-proBNP) and safety indicators (dry cough, symptomatic hypotension, angioedema, etc.). As shown in Table 1.

**TABLE 1:** Characteristics of the clinical trials included in the meta-analysis.

First author (Year) refs	Study design	Intervention and control	Patients (n)	Age	Men (%)	Indices	FU (mo)
Wang (30)	RCT	ARNI/ARB	80/80	59.0 ± 10.3/58.0 ± 10.4	86.25/83.75	➀➂➃➅➆➇➈⑫	6
Yang (31)	RCT	ARNI/ARB	42/45	67.2 ± 4.2/67.6 ± 3.8	59.5/57.8	➀➄➅➆➇➈➉	12
She (26)	Cohort study	ARNI/ARB	259/173	61.82 ± 11.90/62.13 ± 12.53	76.6/77.7	➆➇➈➉⑪	6
Abdelnabi (27)	RCT	ARNI/ARB	45/45	58.0 ± 11.6/59.60 ± 11.6	66.7/64.4	➀➆➇➉⑪	24
Yang (28)	RCT	ARNI/ARB	38/38	60.29 ± 12.71/55.42 ± 11.78	81.6/92.1	➀➂➃➄➆➇➈➉⑪⑫	3
Cui (25)	RCT	ARNI/ARB	102/98	63.74 ± 9.53/62.73 ± 9.87	72.5/70.4	➀➁➅➆➇➈⑫	6
Dong (29)	RCT	ARNI/ARB	40/40	63.9 ± 8.2/62.0 ± 7.6	57.5/65.0	➀➁➅➆➇➈⑪	6
Han (24)	Cohort study	ARNI/ARB	26/48	62.5 ± 12.2/58.0 ± 12.10	69.0/67.0	➀➂➃	6
Ye (32)	Cohort study	ARNI/ARB	84/86	62.29 ± 12.82/63.49 ± 11.61	61.9/65.12	➀➁➈	12

➀ LVEF; ➁ left ventricular end-diastolic diameter (LVEDD); ➂ LVEDV; ➃ left ventricular end-systolic volume (LVESV); ➄ Lad; ➅ NT-ProBNP; ➆ MACE; ➇ HF; ➈ Readmission; ➉ Cardiac Death; ⑪ MI; ⑫ Adverse side effect.

## Quality assessment

**FIGURE 2:**
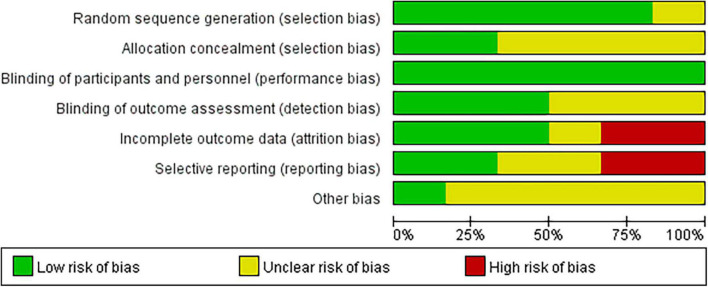
Summary of the quality assessment by The Cochrane Collaboration’s tool.

The Cochrane Collaboration’s tool (22) was used by two reviewers (P. Y. and X.-L.W.) to independently evaluate the RCTs for potential bias. The overall risk of bias was divided into “high risk,” “unclear,” or “low risk” (Figure 2). Qualities of the included cohort studies were evaluated by the Newcastle-Ottawa Assessment Scale (NOS) (23). A high-quality study had a NOS score > 6. As fewer than 10 studies were included, no funnel plot was drawn.

## Statistical analysis

RevMan5.4 software was used to synthesize and analyze the extracted data. Dichotomous variables were reported as Proportions estimated by risk ratio (RR) with 95% confidence interval (CI), and continuous variables were primarily expressed as mean ± *SD* estimated by mean difference (MD) or standard mean difference (SMD) with 95% confidence interval (CI). Forest plots and the *I*^2^-value were used to investigate the heterogeneity between studies. Statistically significant results were identified as CIs excluding a null effect and *P* < 0.05. Heterogeneity between studies was assessed using the Q statistic, and its extent was calculated by the *I*^2^-test, to determine if variability between studies resulted from heterogeneity or chance. If the test showed *I*^2^ > 50%, data had high heterogeneity. The effect of each study on the overall effect size was assessed by a sensitivity analysis using the leave-one-out approach.

## Results

### Search results and baseline characteristics

The search identified 790 articles and 2 studies registered at Clinicaltrials.gov that met the inclusion criteria. After removing duplicates and screening, 9 studies (24–32) involving 1,369 participants were ultimately eligible for analysis.

The characteristics of the included studies are presented in Table 1. Of the 9 studies, 6 were RCTs and 3 were non-RCTs. Based on our defined outcomes, 8 studies reported on CRR outcomes (24, 25, 27–32); 4 studies reported on biomarkers outcomes (25, 29–31); 7 studies reported on MACE and HF outcomes (25–31); 6 studies reported on readmission outcomes (25, 26, 28, 30–32); 4 studies reported on cardiac death outcomes (26–28, 31); 4 studies reported on MI outcomes (26–29); while 3 studies reported on adverse side effects (25, 28, 30).

### Effects of angiotensin-receptor neprilysin inhibitor on cardiac reverse remodeling Indices

#### LVEF

**FIGURE 3:**
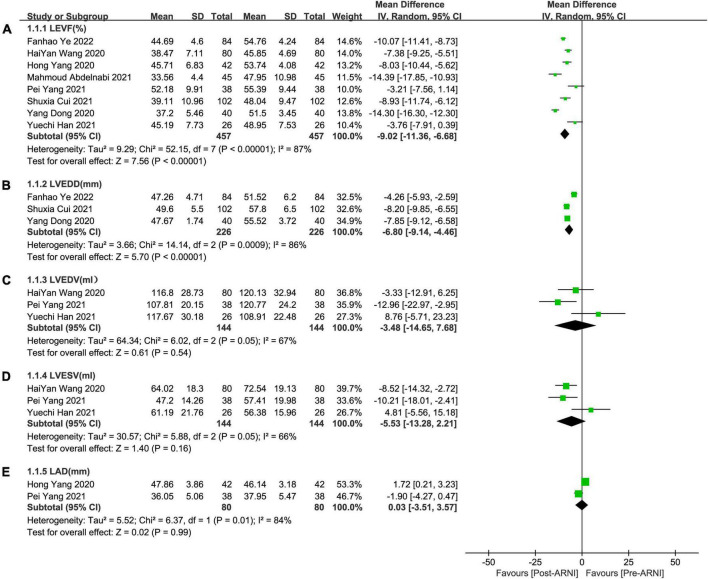
Forest plots for effects of ARNI on LVEF and other CRR indices of myocardial infarction patients. **(A)** The forest plots of the left ventricular ejection fraction, **(B)** the forest plots of the left ventricular end-diastolic diameter, **(C)** the forest plots of the left ventricular end-diastolic volume, **(D)** the forest plots of the left ventricular end-systolic volume, and **(E)** the forest plots of the left atrial dimension.

**FIGURE 4:**
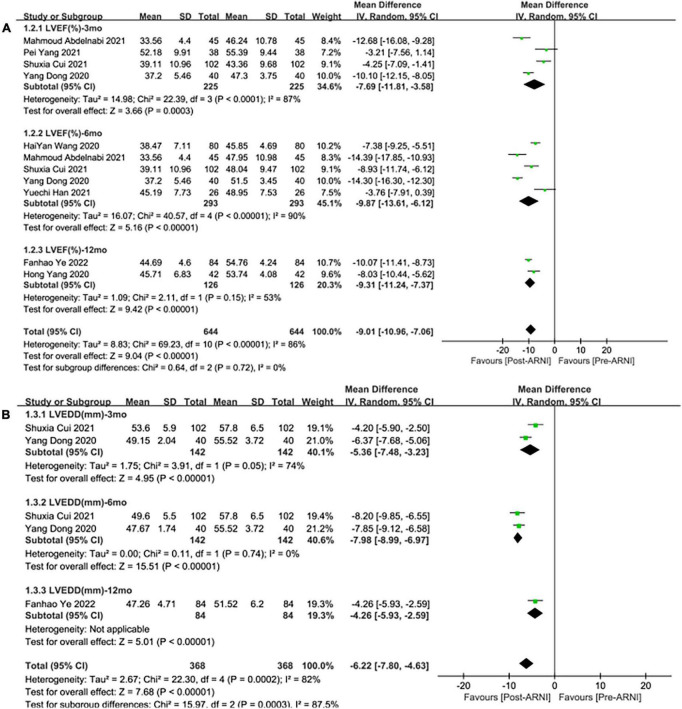
Subgroup analysis of ARNI effects on CRR indices according to follow-up periods (1). **(A)** Subgroup analysis on left ventricular ejection fraction, **(B)** subgroup analysis on left ventricular end-diastolic diameter.

Pooled data involving 937 patients from 8 studies (24, 25, 27–32) showed that LVEF scores increased significantly compared with baseline after treatment with ARNI (MD: 9.02%, [95%CI: 6.68, 11.36]; Figure 3A). Nonetheless, the *I*^2^-value for studies assessing changes was 87%, implying significant heterogeneity across the studies. Subgrouping based on publication year, studies, and follow-up duration had no pronounced effect on the *I*^2^-value. Subgroup analysis showed that LVEF increased significantly in different follow-up times (3, 6, and 12 months), respectively (MD: 7.69%, 95%CI: 3.58, 11.81; Figure 4A), (MD: 9.87%, [95%CI: 6.12, 13.61]; Figure 4A) and (MD: 9.31%, [95%CI: 7.37, 11.24]; Figure 4A), and *I*^2^ was reduced to 0.49 after excluding data from the three studies with higher weights. This heterogeneity may be partly attributable to outcome assessment and reliance on physician judgment. The evaluation criteria for different evaluation methods may likewise vary from study to study.

**FIGURE 5:**
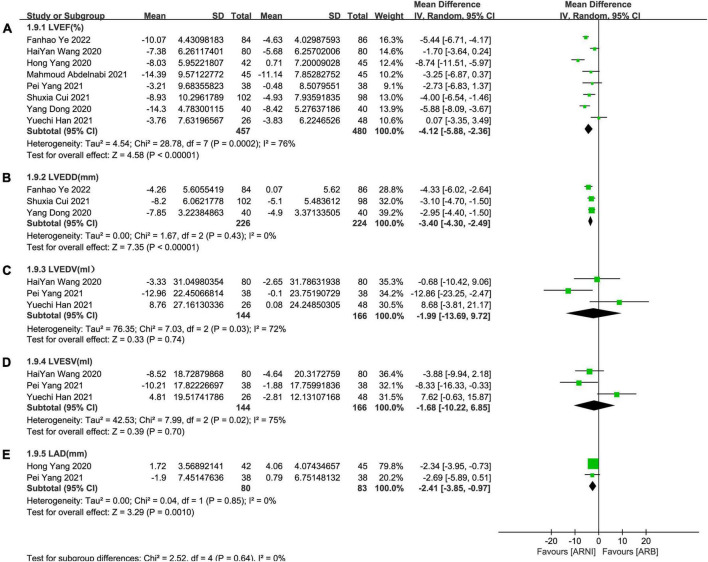
Forest plots for the effect of ARNI on CRR indices in contrast with ARBs. **(A)** The forest plots of the left ventricular ejection fraction, **(B)** the forest plots of the left ventricular end-diastolic diameter, **(C)** the forest plots of the left ventricular end-diastolic volume, **(D)** the forest plots of the left ventricular end-systolic volume, and **(E)** the forest plots of the left atrial dimension.

**FIGURE 6:**
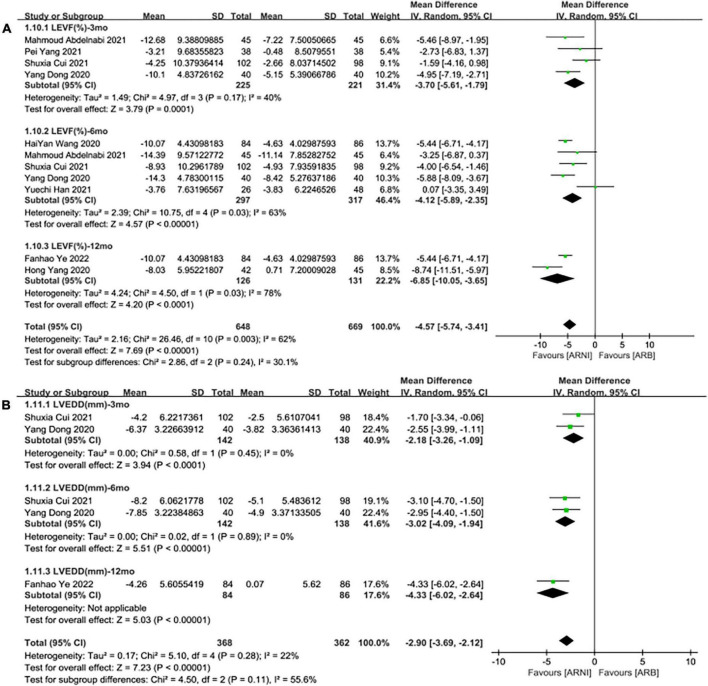
Subgroup analysis of ARNI effects on CRR indices in contrast with ARBs according to follow-up periods (1). **(A)** Subgroup analysis on left ventricular ejection fraction, **(B)** subgroup analysis on left ventricular end-diastolic diameter.

In contrast to ARBs, the improvement effect of the ARNI group was more obvious in LVEF (MD: 4.12%, [95%CI: 2.36, 5.88]; Figure 5A). After the exclusion of 3 studies with high heterogeneity, *I*^2^ was reduced to 0.06. Subgroup analysis based on follow-up times showed that LVEF elevation of ARNI was more effective than that of ARBs (MD: 3.70%, [95%CI: 1.79, 5.61]; Figure 6A), (MD: 4.12%, [95%CI: 2.35, 5.89]; Figure 6A), and (MD: 6.85%, [95%CI: 3.65, 10.05]; Figure 6A) respectively.

#### Left ventricular end-diastolic diameter

Summary data of the three studies (25, 29, 32) showed that left ventricular end-diastolic diameter (LVEDD) decreased significantly compared with baseline after treatment with ARNI (MD: –6.80 mm, [95%CI: –9.14, –4.46]; Figure 3B). Subgroup analysis showed that LVEDD decrease significantly in different follow-up times (3, 6, and 12 months), respectively (MD: –5.36 mm, 95%CI: –7.48, –3.23; Figure 4B), (MD: –7.98 mm, 95%CI: –8.99, –6.97; Figure 4B) and (MD: –4.26 mm, 95%CI: –5.93, –2.59; Figure 4B). Additionally, in contrast to ARBs, ARNI showed a significant difference in LVEDD decrease (MD: –3.40 mm, [95%CI: –4.30, –2.49]; Figure 5B) and the subgroup analysis was also shown in Figure 6B.

#### LVEDV

**FIGURE 7:**
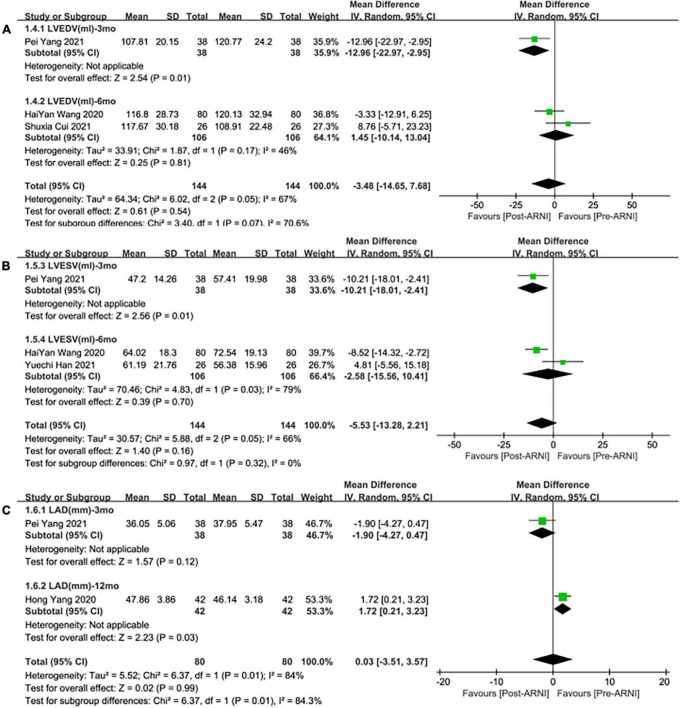
Subgroup analysis of ARNI effects on CRR indices according to follow-up periods (2). **(A)** Subgroup analysis on left ventricular end-diastolic volume, **(B)** subgroup analysis on left ventricular end-systolic volume, and **(C)** subgroup analysis on left atrial dimension.

**FIGURE 8:**
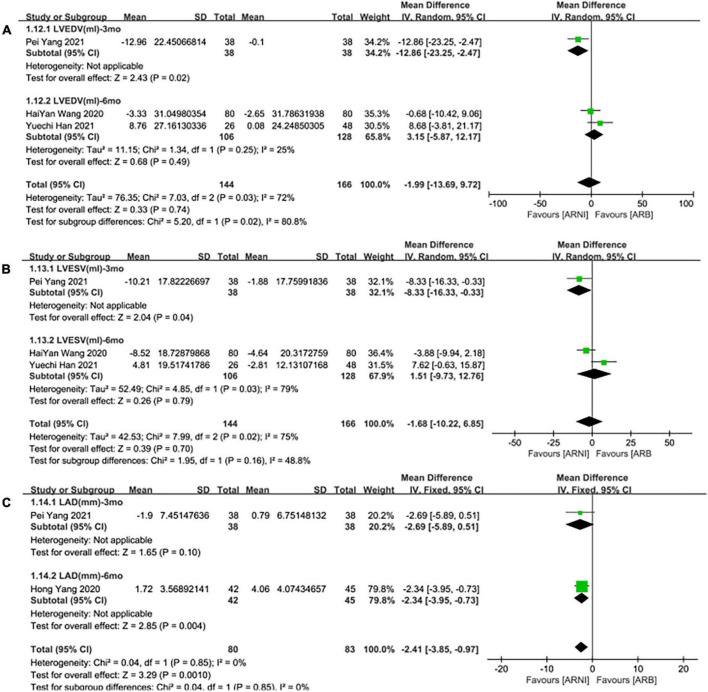
Subgroup analysis of ARNI effects on CRR indices in contrast with ARBs according to follow-up periods (2). **(A)** Subgroup analysis on left ventricular end-diastolic volume, **(B)** subgroup analysis on left ventricular end-systolic volume, and **(C)** subgroup analysis on left atrial dimension.

Three studies (24, 28, 30) involving 144 patients showed that there was no statistically significant in LVEDV change compared with baseline after treatment with ARNI (MD: –3.48 mL, [95%CI: –14.65, 7.68]; Figure 3C). But in fact, subgroup analysis based on follow-up times (3, 6 months) showed different results (MD: –12.96 ml, 95%CI: –22.97, –2.95; Figure 7A) and (MD: –1.45 ml, 95%CI: –10.14, 13.04; Figure 7A), respectively, which indicated a point in dispute. Moreover, there was no significant difference in LVEDV reduction with ARNI compared with ARB (MD: −1.99 ml, [95%CI: −13.69, 9.72]; Figure 5C) and the subgroup analysis also came to the same results, Figure 8A.

#### Left ventricular end-systolic volume

Summary data from three studies (24, 28, 30) showed that there was no significant difference in left ventricular end-systolic volume (LVESV) decrease after taking ARNIcompared with baseline (MD: –5.53 ml, [95%CI: –13.28, 2.21]; Figure 3D). Subgroup analysis based on follow-up showed significant difference in LVESV decrease at 3 months (MD: –10.21 ml, [95%CI: –18.01, –2.41]; Figure 7B), and no significant at 6 months follow-up (MD: –2.58 mL, [95%CI: –15.56, 10.41]; Figure 7B).

It showed that there was no significant difference in LVESV decrease after taking ARNI vs. ARBs compared with baseline (MD: –1.68 ml, [95%CI: –10.22, 6.85]; Figure 5D). Subgroup analysis based on follow-up showed significant difference in LVESV decrease at 3 months follow-up (MD: –8.33 ml, [95%CI: –16.33, –0.33]; Figure 8B), and no significant difference in LVESV decrease at 6 months follow-up (MD: 1.51 ml, [95%CI: –9.73, 12.76]; Figure 8B).

#### LAd

Data from two studies (28, 31) showed that there was no significant difference in LAd decrease after taking ARNI compared with baseline (MD: 0.03 mm, [95%CI: –3.51, 3.57]; Figure 3E). Subgroup analysis based on follow-up times showed that there was no significant difference in the decrease of LAd in 3 months follow-up (MD: –1.90 mm, [95%CI: –4.27, 0.47]; Figure 7C), while there was a significant difference in the decrease of LAd in 12 months follow-up (MD: 1.72 mm, [95%CI: 0.21, 3.23]; Figure 7C).

It also showed that there was a significant difference in LAd reduction after taking ARNI vs. ARBs compared with baseline (MD: –2.41 mm, [95%CI: –3.85, –0.97]; Figure 5E). Subgroup analysis based on follow-up showed significant difference in the decrease of LAd in 3 and 6 months, respectively (MD: –2.69 mm, [95%CI: –5.89, 0.51]; Figure 8C) and (MD: –2.34 mm, [95%CI: –3.95, –0.73]; Figure 8C).

### Effects of angiotensin-receptor neprilysin inhibitor on biomarkers

**FIGURE 9:**
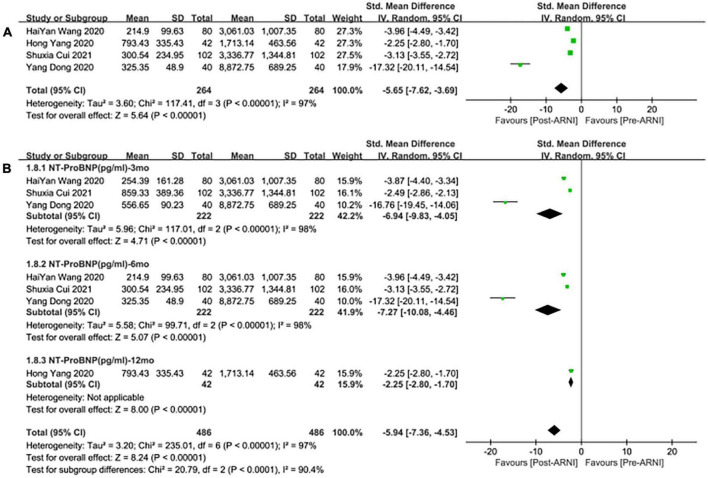
Forest plots for the effect of ARNI on remodeling biomarkers **(A)** and subgroup analysis **(B)**.

Pooled data of 4 studies (25, 29–31) showed a significant difference in NT-proBNP reduced after taking ARNI compared with baseline (SMD: –5.65, [95%CI: –7.62, –3.69]; Figure 9A). Subgroup analysis based on follow-up duration showed that there were significant differences in each follow-up times (3, 6, 12 months), which were (SMD: –6.94, [95%CI: –9.83, –4.05); Figure 9B], (SMD: –7.27, [95%CI: –10.08, –4.46]; Figure 9B), and (SMD: –2.25, [95%CI: –2.80, –1.70]; Figure 9B), respectively.

**FIGURE 10:**
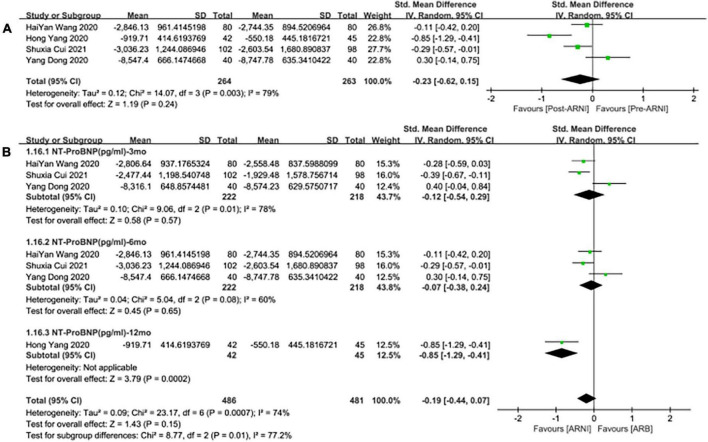
Forest plots for the effect of ARNI on remodeling biomarkers in contrast with ARBs **(A)** and subgroup analysis **(B)**.

Meanwhile, the studies showed that there was no significant difference in the decrease of NT-proBNP by taking ARNI compared with ARB (SMD: –0.23, [95%CI: –0.62, 0.15]; Figure 10A). Subgroup analysis based on follow-up periods showed that there was also no significant difference in the decrease of NT-proBNP in the follow-up of 3 and 6 months, respectively (SMD: –0.12, [95%C: –0.54, 0.29]; Figure 10B), (SMD: –0.07, [95%CI: –0.38, 0.24]; Figure 10B). Whereas, there was a significant difference in the follow-up of NT-proBNP in 12 months (SMD: –0.85, [95%CI: –1.29, –0.41]; Figure 10B).

### MACE

**FIGURE 11:**
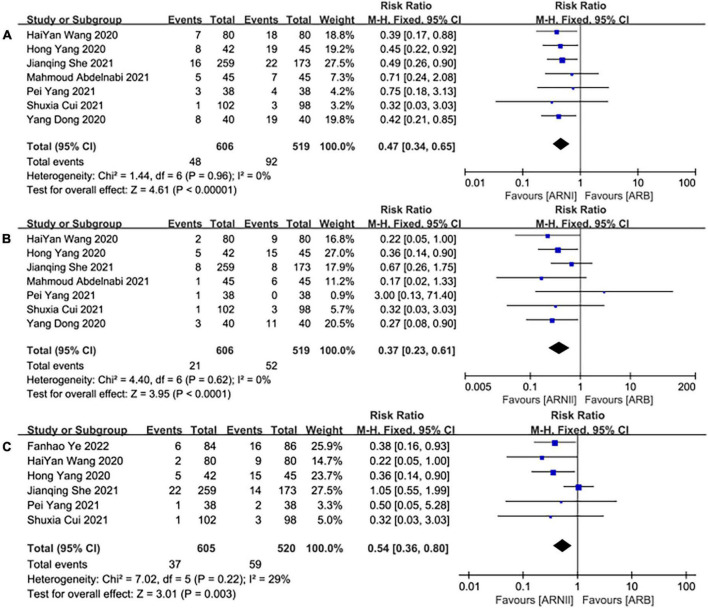
The forest plots of the effectiveness and safety outcomes between early initiation of sacubitril/valsartan and angiotensin receptor blocker in patients after acute myocardial infarction (1). **(A)** The forest plots of the incidence of major adverse cardiac events, **(B)** the forest plots of the incidence of heart failure, and **(C)** the forest plots of the readmission.

Seven studies (25–31) involving a total of 1,125 patients reported MACE outcomes. There was no significant heterogeneity among the included studies (*I*^2^ = 0.0%, *P* = 0.96). Therefore, the fixed effects M-H model was used. The meta-analysis showed that the incidence of MACE in the ARNI group was lower than that in the ARBs (RR: 0.47, [95%CI: 0.34, 0.65, *P* < 0.00001]; Figure 11A).

### HF

Seven studies (25–31) involving a total of 1,125 patients reported HF outcomes. There was no significant heterogeneity among the included studies (*I*^2^ = 0.0%, *P* = 0.62). Therefore, the fixed effects M-H model was used. The meta-analysis showed that the incidence of HF in the ARNI group was lower than that in the ARBs (RR: 0.37, [95%CI: 0.23, 0.61, *P*<0.00001]; Figure 11B).

### Readmission

Six studies (25, 26, 28, 30–32) involving 1,125 patients reported readmission outcomes. There was no significant heterogeneity among the included studies (*I*^2^ = 29%, *P* = 0.22). In consequence, the fixed effects M-H model was used. The meta-analysis showed that the incidence of readmission in the ARNI group was lower than that of the ARB group (RR: 0.54, [95%CI: 0.36, 0.80, *P* = 0.003]; Figure 11C).

### Cardiac death

**FIGURE 12:**
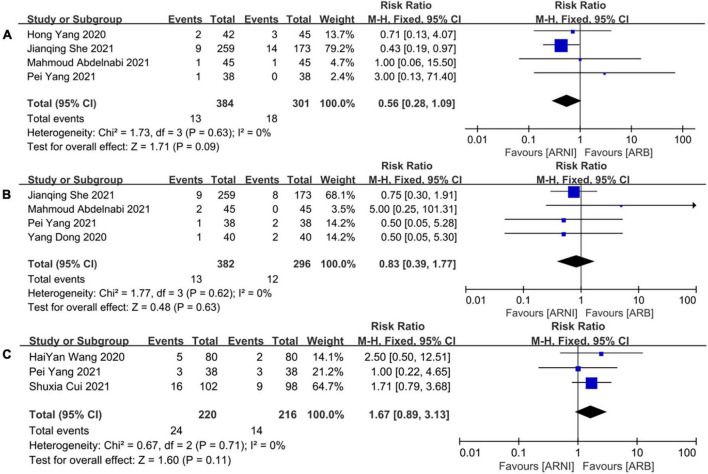
The forest plots of the effectiveness and safety outcomes between early initiation of sacubitril/valsartan and angiotensin receptor blocker in patients after acute myocardial infarction (2). **(A)** The forest plots of the incidence of cardiac death, **(B)** the forest plots of the incidence of myocardial infarction, and **(C)** the forest plots of the adverse side effects.

A total of 685 patients reported cardiac death outcomes in 4 trials (26–28, 31). There was no significant heterogeneity among the included studies (*I*^2^ = 0%, *P* = 0.63). Hence, the fixed effects M-H model was used. The meta-analysis showed that there was no significant difference in the incidence of cardiac death between the two groups (RR: 0.56, [95%CI: 0.28, 1.09, *P* = 0.09]; Figure 12A).

### MI outcome

A total of 678 patients reported MI outcomes in 4 trials (26–29). There was no significant heterogeneity among the included studies (*I*^2^ = 0%, *P* = 0.62). Thus, the fixed effects M-H model was used. The meta-analysis showed that there was no significant difference in the incidence of cardiac death between the two groups (RR: 0.83, [95%CI: 0.39, 1.77, *P* = 0.63]; Figure 12B).

### Adverse side effects

A total of 436 patients reported adverse outcomes in 3 trials (25, 28, 30). There was no significant heterogeneity among the included studies (*I*^2^ = 0%, *P* = 0.71). Therefore, the fixed effects M-H model was used. The meta-analysis showed that there was no significant difference in the incidence of adverse reactions between the ARNI group and the ARB group (RR: 1.67, [95%CI: 0.89, 3.13, *P* = 0.11]; Figure 12C).

## Discussion

Since the advent of sacubitril/valsartan, its benefits for HF patients have been confirmed in most studies (33–36) Considering that sacubitril/valsartan inhibits both natriuretic peptide system degradation and RAAS system, the significant efficacy of sacubitril/valsartan is currently derived only from patients with heart failure, many investigators speculate that sacubitril/valsartan has benefits in patients with AMI, but the benefits and risks remain controversial (9, 15, 37, 38). Recently, Zhao et al. (13) found that sacubitril/valsartan was superior to ACEI in reducing the risks of major adverse cardiac events and left ventricular ejection fraction increasing. Whereas, Sacubitril/Valsartan showed no obvious advantage over ACEI in the outcomes (the incidences of cardiac death, heart failure hospitalization, myocardial infarction, and adverse side effects).

However, another meta-analysis (15) also points out the controversy from Docherty et al. (38) found that in patients with asymptomatic LV systolic dysfunction late after myocardial infarction, treatment with sacubitril/valsartan did not have a significant reverse remodeling effect compared with valsartan.

Therefore, nine studies involving 1,369 patients were included in this meta-analysis to evaluate the efficacy and safety (including CRR indices, biological markers, MACE, etc.) of ARNI in patients with myocardial infarction after interventional treatment.

We not only compared data before and post ARNI treatment but also analyzed ARNI and ARB indices. The results showed that LVEF, LVEDD, and NT-proBNP indices were significantly ameliorated after ARNI treatment. Meanwhile, compared with ARB treatment, the indices of LVEF, LVEDD, and LAd of patients treated with ARNI were significantly ameliorated. When studies were restricted to those ejection fraction < 40%, indices (LVEF, LVEDD) were also significantly changed, (MD: 11.18%, [95%CI: 7.33, 15.03]) and (MD: –7.98 mm, [95%CI: –8.99, –6.97]). Subgroup analysis showed that there were significant differences in the indices of ARNI compared with ARB in different follow-up times (3, 6, and 12 months), suggesting that the treatment effects of ARNI were better than that of ARB starting 3 months and keeping. The short-term benefit of ARNI on CRR may be related to its long-term effect on cardiovascular outcomes. Early treatment with ARNI in eligible patients may be beneficial.

Similarly, short-term use of ARNI had a significant effect on NT-proBNP levels in these patients with myocardial infarction or those with ejection fraction <40%. But meta-analysis showed high heterogeneity in ARNI or ARB. The occurrence of this situation may be caused by the inconsistencies of the degree of illness caused by the infarct area, visit time of myocardial infarction patients (39), or other factors, resulting in a large difference in the level of NT-proBNP, resulting in uneven baseline variance, or caused by inappropriate original data analysis methods. However, with the extension of follow-up time, heterogeneity gradually decreased. According to the current results, early taking ARNI in these patients can significantly decrease the level of NT-proBNP, and compared with ARB, long-term use may have greater benefits.

After treatment, the incidence of MACE, heart failure rate, and readmission rate in the ARNI group were significantly lower than those in the ARB group, and the results were consistent with expectations. The study found that one of the cohort studies had a relatively large sample size and a relatively long follow-up time in terms of safety (26). Therefore, when RCT studies were included only, the meta-analysis MACE (RR: 0.46, [95%CI: 0.32, 0.68, *P* < 0.0001]) and heart failure (RR: 0.31, [95%CI: 0.17, 0.55, *P* < 0.0001]) and readmission (RR: 0.32, [95%CI: 0.16, 0.65, *P* = 0.002]). Although RCTs have strict inclusion criteria, some patients with weakness may be excluded from RCT before randomization, and the follow-up time is short, resulting in a relatively small sample size of patients. However, when all studies are included, there is no obvious contradiction between the same results and the same results, which increases the reliability of results while enlarging the sample size. Similarly, When studies were restricted to those ejection fraction <40%, indices (MACE, HF, readmission) were also lower than those in the ARB group, MACE (RR: 0.45, [95%CI: 0.28, 0.71, *P* = 0.0007]), HF (RR: 0.24, [95%CI: 0.11, 0.53, *P* = 0.0005]), and readmission (RR: 0.25, [95%CI: 0.07, 0.86, *P* = 0.002]). Therefore, the current meta-analysis results are generally reliable.

### Limitations

This study has several limitations: some studies reported some results in advance, which may affect the overall quality of the study (27). Therefore, these results should be interpreted with caution; only 9 studies were included in the comparison of ARNI and ARB, a limited number of which may be due to the fact that ARNI was rarely used in patients with AMI at present. ARBs have, in fact, seldom been used as the control group in these papers. Perhaps related studies are ongoing or the results have not been published. A small number of patients were included in some studies, and sequence generation and allocation hiding were not reported in most of the included studies, which may lead to selection bias. Hence, results may be affected by unpredictable factors.

## Conclusion

This meta-analysis revealed that patients with AMI receiving ARNI treatment as early as possible and lasting at least 3 months may benefit more from CRR and MACE risk than patients with ARB. Further studies are still needed to explore the long-term effects of ARNI on AMI patients and clarify the relationship between short-term CRR and long-term clinical outcomes to support doctors’ ability to make an early prognosis.

## Data availability statement

The datasets presented in this study can be found in online repositories. The names of the repository/repositories and accession number(s) can be found in the article/Supplementary material.

## Author contributions

PY, YH, CL, and XW contributed to revising the work critically. All authors have approved the final version to be published and have agreed to be accountable for all aspects of the work, substantially to the conception and design of the work, acquisition, interpretation of data, and drafted the work.
