# Supplementary figures and images for: Efficacy and safety of sacubitril/valsartan vs. valsartan in patients with acute myocardial infarction: A meta-analysis
Source: Front Cardiovasc Med. 2022 Aug 24;9:988117. doi: 10.3389/fcvm.2022.988117 (PMC9448932; doi:10.3389/fcvm.2022.988117)

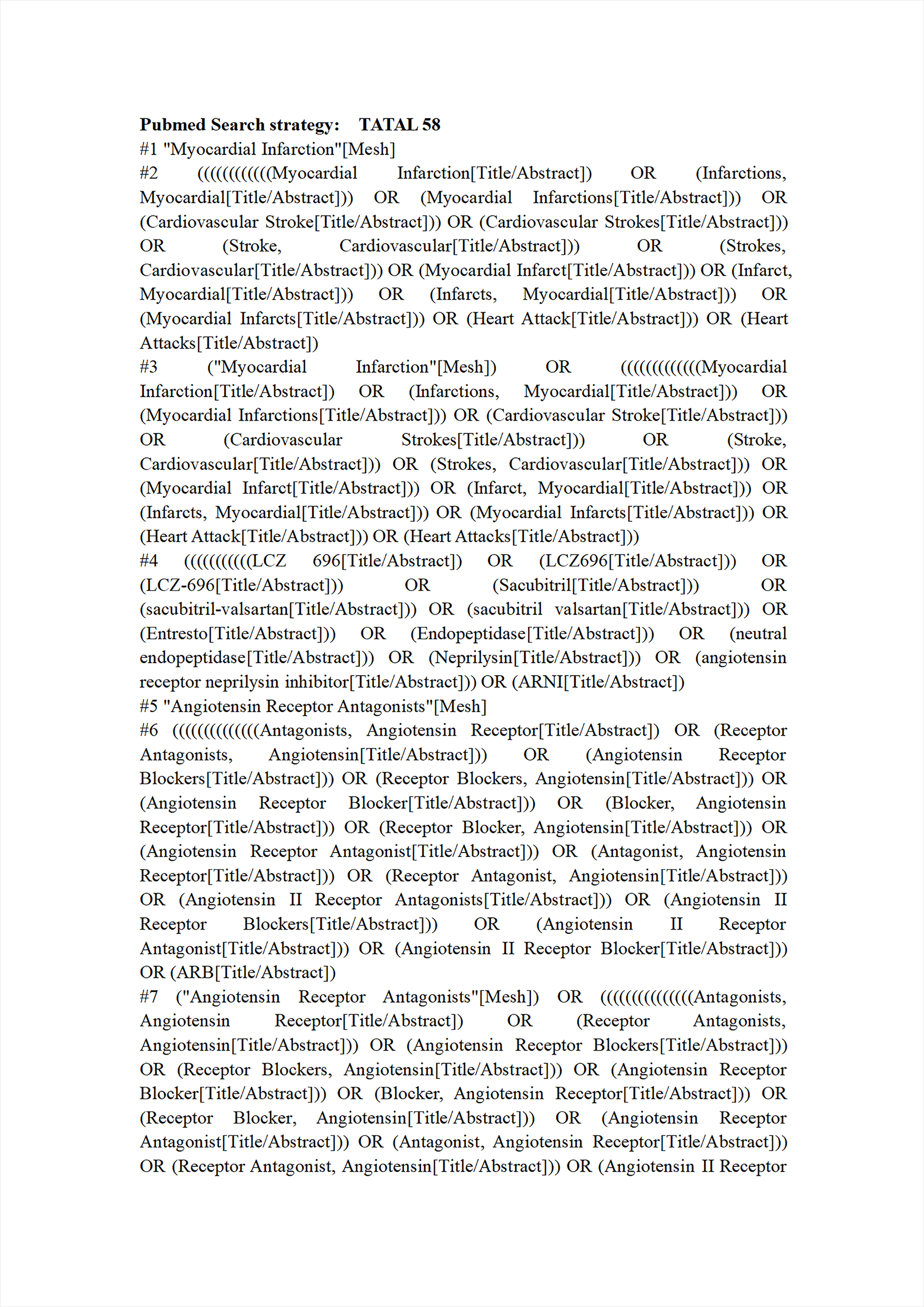

Supplement: Supplementary file 1 [file Image_1.TIF]

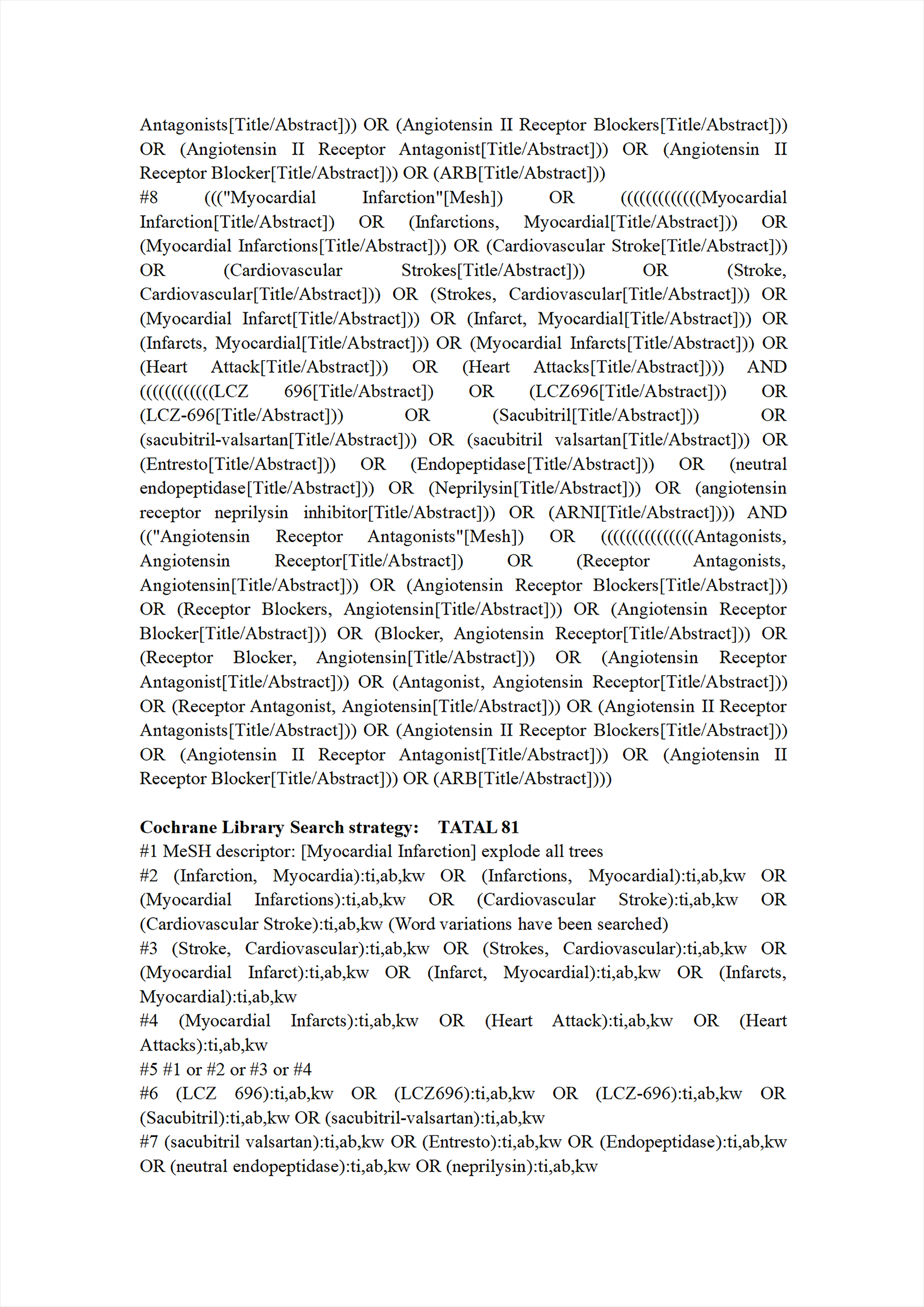

Supplement: Supplementary file 2 [file Image_2.TIF]

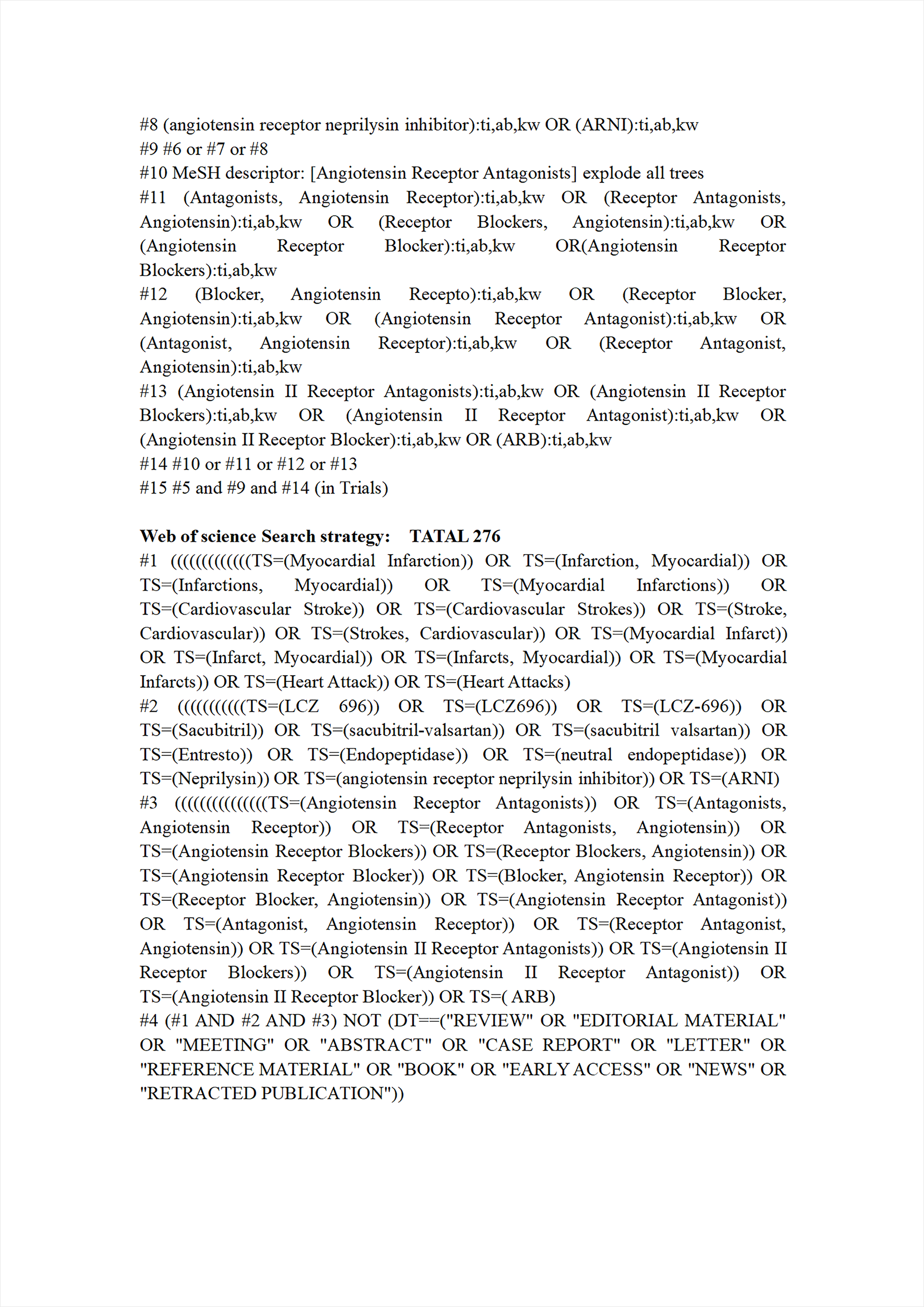

Supplement: Supplementary file 3 [file Image_3.TIF]

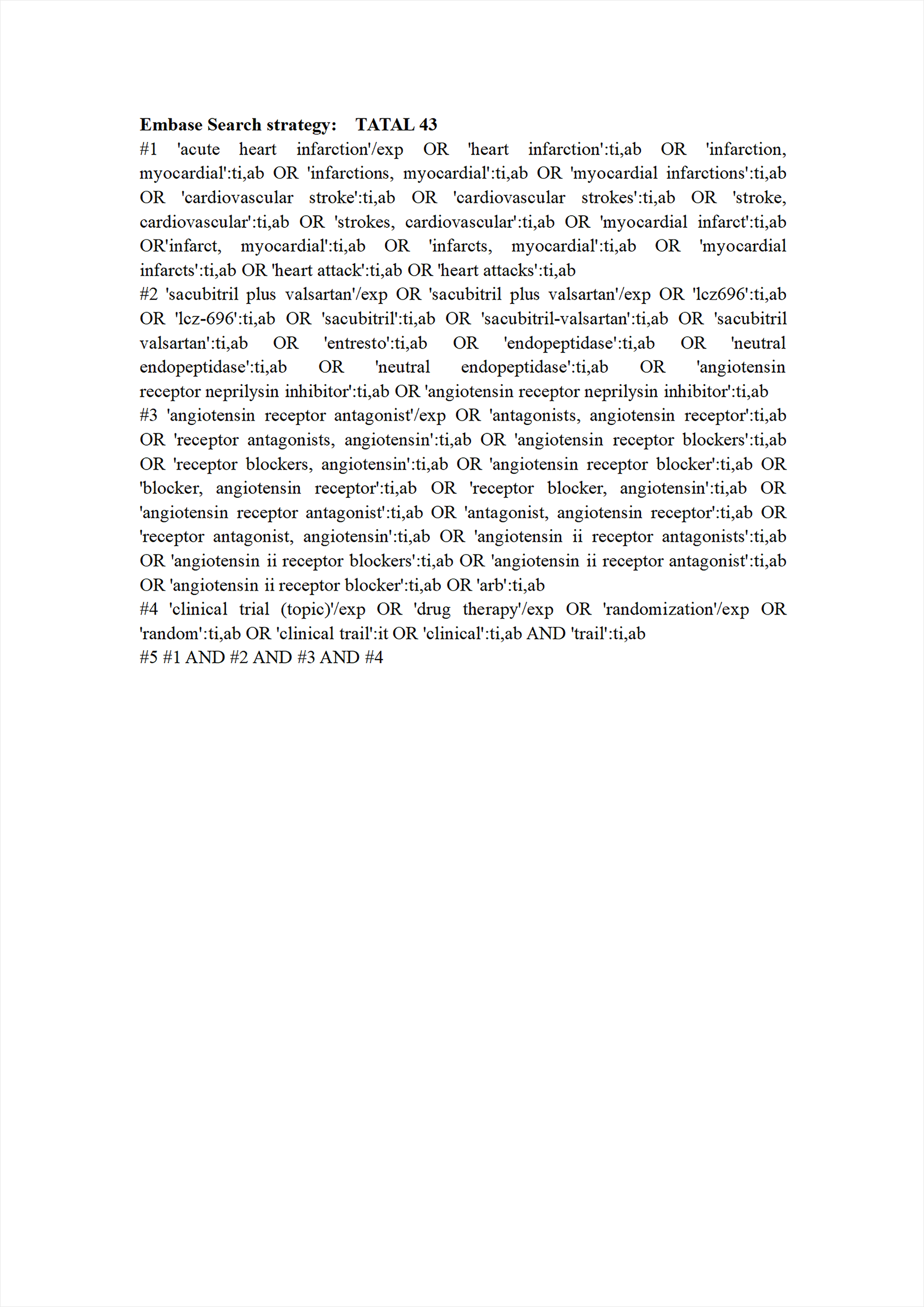

Supplement: Supplementary file 4 [file Image_4.TIF]
